# Preprocedural CT Radiomic Features of the Basal Ganglia Are Associated with Post-Thrombectomy Hyperdense Transformation: A LASSO-Based Logistic Regression Analysis

**DOI:** 10.3390/tomography12100149

**Published:** 2026-10-08

**Authors:** Ahmet Bayrak, Uğur Kesimal, Şahap Törenek, Yasin Özdemir, Erkan Bilgin, Çetin İmamoğlu, Ezel Yaltırık Bilgin

**Affiliations:** 1 Department of Radiology, Ankara Dr. Abdurrahman Yurtaslan Oncology Training and Research Hospital, Ankara 06200, Turkey; sahap.torenek@hotmail.com (Ş.T.); ozdemiryasin181818@gmail.com (Y.Ö.); erk314@yahoo.com (E.B.); cetinimamoglu@gmail.com (Ç.İ.); ezelyaltirik@yahoo.com (E.Y.B.); 2 Department of Radiology, Ankara Training and Research Hospital, Ankara 06230, Turkey; ugur_kesimal@hotmail.com

**Keywords:** acute ischemic stroke, mechanical thrombectomy, radiomics, basal ganglia, hyperdense transformation

## Abstract

**Purpose:** To investigate whether radiomic characteristics of visually unremarkable basal ganglia on preprocedural non-contrast computed tomography (CT) are associated with subsequent post-thrombectomy hyperdense transformation (HT) in patients with acute ischemic stroke. **Materials and Methods:** This retrospective single-center study included a derivation cohort of 84 patients with preserved Alberta Stroke Programme Early CT Scores (ASPECTS ≥ 8), no visually appreciable basal ganglia abnormality, anterior circulation large-vessel occlusion, and complete reperfusion after mechanical thrombectomy between January 2022 and July 2023. An independent temporal validation cohort comprised 31 consecutive eligible patients treated between August and November 2023. The ipsilateral basal ganglia were manually segmented on preprocedural non-contrast CT, and radiomic features were extracted using LIFEx. Candidate features underwent interobserver reproducibility assessment and correlation filtering before least absolute shrinkage and selection operator logistic regression with 10-fold cross-validation. The final model was applied without modification to the temporal validation cohort. Nested cross-validation and learning-curve analysis were additionally performed to assess model-development optimism and stability. **Results:** The final model retained two texture features, Gray-Level Run Length Matrix Short Runs Emphasis and Gray-Level Size Zone Matrix Grey Level Variance. In the derivation cohort, the model achieved an area under the receiver operating characteristic curve (AUC) of 0.760 (95% CI, 0.652–0.855), with 48.8% sensitivity, 95.3% specificity, and 72.6% accuracy. When applied without modification to the independent temporal validation cohort, the AUC was 0.677 (95% CI, 0.475–0.858), with 33.3% sensitivity, 93.8% specificity, and 64.5% accuracy. Nested cross-validation yielded a mean AUC of 0.673 ± 0.037, with variability in feature selection across resampling iterations. Learning-curve analysis showed an early plateau, with little improvement in cross-validated discrimination as the training sample increased within the evaluated range. **Conclusions:** Radiomic analysis of visually unremarkable basal ganglia on preprocedural non-contrast CT identified an exploratory two-feature imaging signature associated with subsequent post-thrombectomy HT. The model retained modest discriminatory ability in temporal validation, although internal resampling demonstrated model-development optimism and limited feature-selection stability. These findings should be considered hypothesis-generating and warrant further evaluation using larger datasets, alternative modeling approaches, and independent multicenter validation.

## Simple Summary

Patients with acute ischemic stroke may develop new areas of increased density in the brain on computed tomography (CT) after successful mechanical thrombectomy. These changes may reflect contrast leakage or bleeding related to tissue injury following restoration of blood flow. In this study, we investigated whether subtle quantitative characteristics of the basal ganglia on CT scans obtained before thrombectomy were associated with the subsequent development of these imaging changes, even when the basal ganglia appeared normal on visual assessment. We identified two quantitative imaging features that were associated with post-thrombectomy hyperdense transformation. The resulting model showed modest ability to distinguish patients who developed these changes from those who did not, although its performance was lower when tested in a separate group of patients. These findings suggest that apparently normal brain tissue on routine CT may contain subtle quantitative information related to its response after blood flow is restored. However, the results are preliminary and require confirmation in larger studies involving multiple centers before they can be considered for clinical use.

## 1. Introduction

Mechanical thrombectomy has become the standard treatment for acute ischemic stroke caused by large-vessel occlusion and has substantially improved clinical outcomes through rapid restoration of cerebral blood flow [1,2,3,4]. However, reperfusion-related imaging abnormalities remain common despite successful recanalization. Among these, post-procedural intracranial hyperdense transformation (HT)—including contrast extravasation and hemorrhagic transformation—is frequently observed on early follow-up computed tomography (CT) examinations [5,6,7,8,9].

Post-thrombectomy hyperdensity may reflect blood–brain barrier disruption and microvascular injury occurring in the setting of ischemia and subsequent reperfusion. Although contrast extravasation and hemorrhagic transformation represent distinct radiological and clinical entities, both may occur within the broader spectrum of reperfusion-related tissue alterations and can be difficult to distinguish on early non-contrast CT. Several clinical, imaging, and procedural factors have been associated with hemorrhagic complications after reperfusion therapy; however, the extent to which preprocedural CT contains subtle quantitative information associated with subsequent tissue response remains incompletely understood [10,11,12,13,14,15,16,17].

Radiomics enables the extraction of quantitative imaging features that may characterize image heterogeneity and tissue characteristics not appreciable by conventional visual assessment [18,19]. Radiomics-based approaches have increasingly been investigated in stroke imaging, including for characterization of infarct evolution, treatment response, and hemorrhagic complications [20,21]. Such techniques may therefore provide a means of investigating whether brain tissue that appears morphologically unremarkable on baseline CT contains quantitative imaging signatures associated with its subsequent response to reperfusion.

The basal ganglia represent one of the most vulnerable regions of the brain to ischemic injury [10,22,23,24,25]. This vulnerability has been attributed to several anatomical and physiological characteristics, including high metabolic demand, dependence on terminal perforating arterial circulation with limited collateral supply, and susceptibility to blood–brain barrier disruption during reperfusion [26,27,28,29,30,31,32]. Consequently, hemorrhagic transformation and post-thrombectomy hyperdense appearance are frequently encountered within deep gray matter structures, particularly at the level of the basal ganglia [8,33,34,35,36,37,38,39,40]. Importantly, patients undergoing thrombectomy may present with preserved Alberta Stroke Programme Early CT Scores (ASPECTS) and no visually appreciable abnormalities within the basal ganglia on baseline non-contrast CT, despite the possibility of subtle tissue alterations related to ischemia and early microvascular injury. Radiomic analysis may provide a means of detecting quantitative imaging characteristics within such visually unremarkable tissue beyond conventional visual assessment.

Based on these pathophysiological considerations, we hypothesized that radiomic characteristics of visually unremarkable basal ganglia on preprocedural non-contrast CT may be associated with subsequent post-thrombectomy HT. Therefore, the aim of this study was to identify an exploratory basal ganglia-based radiomic signature associated with post-thrombectomy HT in patients with preserved ASPECTS and to assess its robustness through internal resampling and independent temporal validation.

## 2. Materials and Methods

### 2.1. Study Design and Ethical Approval

This retrospective single-center study was approved by the institutional review board (Approval No. 905/2026; 21 May 2026). Due to the retrospective nature of the study, the requirement for informed consent was waived by the institutional review board.

### 2.2. Patient Population

All consecutive patients who underwent mechanical thrombectomy for acute ischemic stroke between January 2022 and July 2023 were retrospectively screened for the derivation cohort, and patients treated between August 2023 and November 2023 were screened for the independent temporal validation cohort. November 2023 represented the end of the study sampling period and corresponded to the end of the investigators’ active involvement in the institutional thrombectomy service. Patients treated thereafter were managed by a different clinical team and were therefore outside the prespecified study cohort.

Only patients with preserved Alberta Stroke Programme Early CT Scores (ASPECTS ≥ 8) and without visually appreciable ischemic changes within the basal ganglia region on baseline CT were included.

Patients were excluded based on predefined criteria, including posterior circulation occlusion, absence of pre- or post-procedural CT imaging, unsuccessful reperfusion (defined as Thrombolysis In Cerebral Infarction [TICI] score < 3), intraprocedural clinical deterioration requiring intensive care unit transfer, and the presence of ipsilateral basal ganglia hypodensity related to a previous stroke, significant gliosis or encephalomalacia, or current acute ischemia that could compromise the reliability of segmentation.

Isolated M2 occlusions were excluded because perfusion of the basal ganglia is typically preserved in many of these patients, potentially limiting the biological relevance of basal ganglia-based radiomic analysis. Therefore, only patients with total internal carotid artery (ICA) occlusion without significant filling through communicating vessels and patients with total M1 occlusion were included.

The study population was intentionally restricted to patients with preserved ASPECTS and no visually appreciable basal ganglia abnormalities on baseline CT. Patients with ASPECTS <8 were excluded to minimize the confounding effects of extensive ischemic injury and infarct burden and to enable evaluation of quantitative imaging characteristics within tissue that appeared unremarkable on conventional imaging.

To avoid double counting, when multiple exclusion criteria applied to a single patient, one primary exclusion reason was assigned using a predefined hierarchical approach.

**Figure 1:**
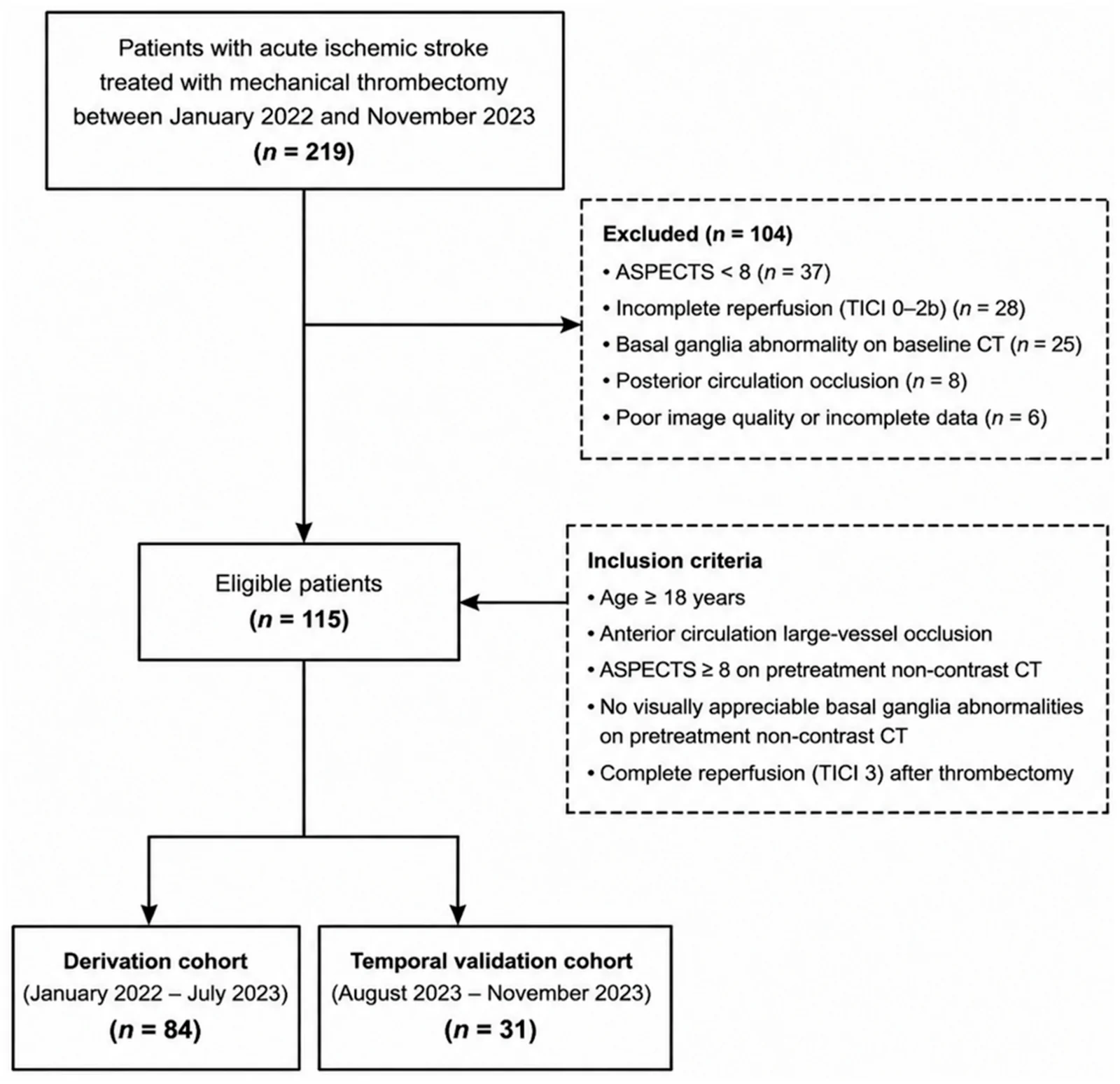
Flowchart of patient selection for derivation and temporal validation cohorts.

After application of the predefined eligibility criteria, 84 of 160 screened patients were included in the derivation cohort. For temporal validation, 31 of 59 consecutively screened patients met the eligibility criteria and were included in the independent validation cohort (Figure 1).

### 2.3. Mechanical Thrombectomy Procedure

Mechanical thrombectomy procedures were performed according to institutional protocols by experienced interventional radiologists. Reperfusion success was assessed using the modified TICI scale. Only patients with complete reperfusion (TICI 3) were included to reduce heterogeneity related to incomplete recanalization. Patients with TICI < 3 reperfusion were excluded because heterogeneous or incomplete reperfusion could confound the relationship between preprocedural tissue characteristics and subsequent reperfusion-related imaging changes.

### 2.4. CT Acquisition and Reconstruction

All patients underwent preprocedural non-contrast cranial CT using the same scanner and acquisition protocol. Source images were acquired using a Toshiba Aquilion CX 128-slice scanner (Canon Medical Systems Corp., Otawara, Tochigi, Japan) at 120 kVp and 200–350 mA, with a nominal acquisition slice thickness of 0.5 mm and a sharp reconstruction kernel (FC51/52). For segmentation and radiomic analysis, axial images reconstructed at a slice thickness of 2.5 mm were used consistently in the derivation and temporal validation cohorts. The same reconstruction approach was used for the interobserver reproducibility analysis.

Post-procedural CT imaging was obtained as part of routine clinical follow-up.

### 2.5. Image Segmentation

**Figure 2:**
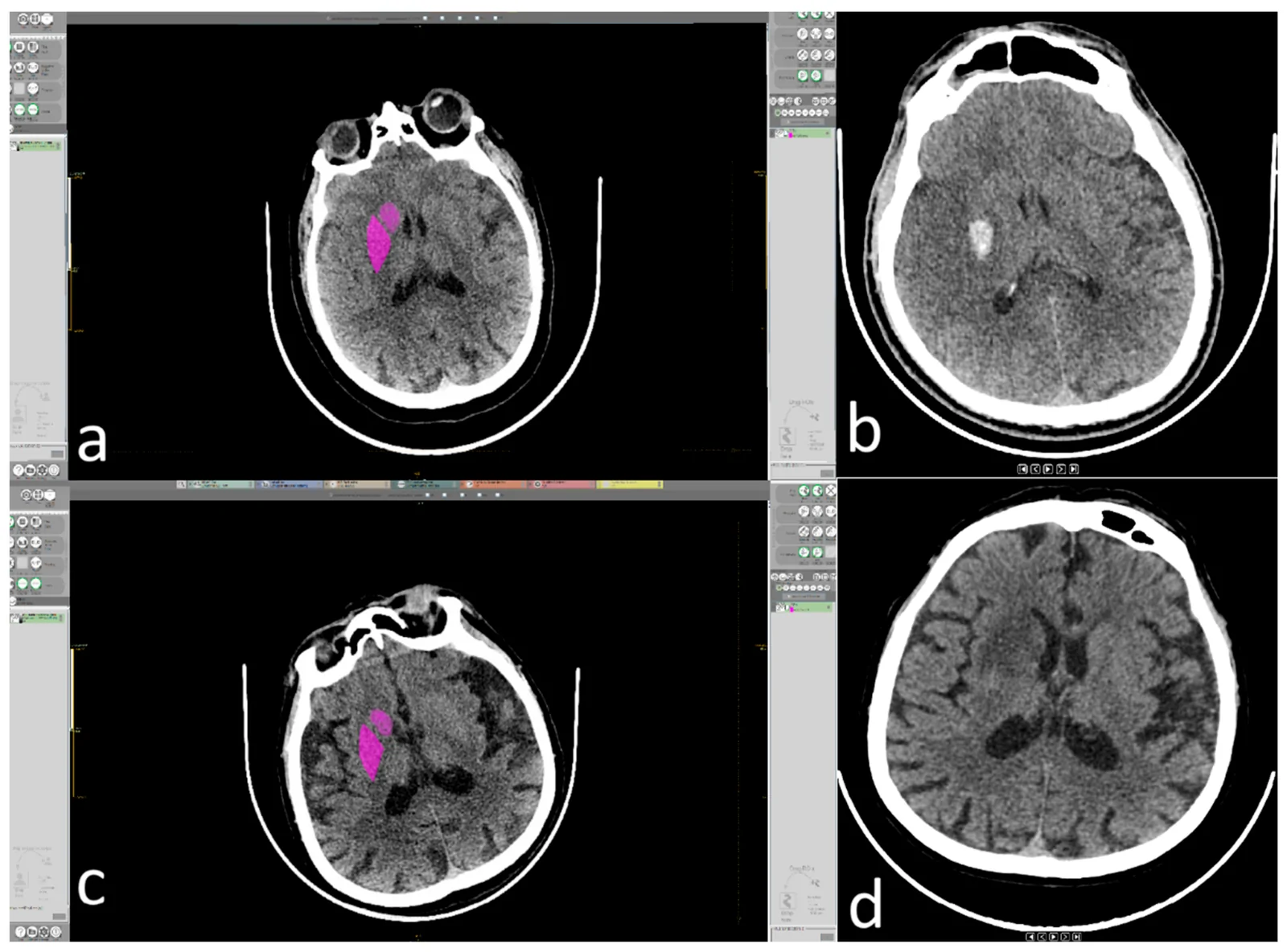
Representative cases of basal ganglia segmentation and post-thrombectomy imaging outcomes. (**a**) Preprocedural non-contrast CT image demonstrating manual segmentation of the ipsilateral basal ganglia in a patient who subsequently developed hyperdense transformation (HT). (**b**) Follow-up CT obtained approximately 24 h after thrombectomy demonstrating HT with intraparenchymal hemorrhage. (**c**) Preprocedural non-contrast CT image demonstrating basal ganglia segmentation in a patient without subsequent HT. (**d**) Follow-up CT obtained approximately 24 h after thrombectomy showing no evidence of HT. The pink overlay in panels (**a**,**c**) indicates the manually segmented basal ganglia VOI. The partially visible interface elements in panels (**a**,**c**) correspond to the LIFEx software used for segmentation.

Manual three-dimensional segmentation of the basal ganglia was performed on the 2.5 mm reconstructed axial preprocedural non-contrast CT images. Consecutive regions of interest (ROIs) were manually delineated on each axial image containing the basal ganglia and combined to generate a three-dimensional volume of interest (VOI). The segmented region included the caudate nucleus, lentiform nucleus, and adjacent internal and external capsules. Care was taken to exclude adjacent vessels, calcifications, and osseous structures to minimize partial-volume effects. Representative examples of segmentation and corresponding post-thrombectomy imaging outcomes are shown in Figure 2.

Only the basal ganglia ipsilateral to the target vessel occlusion were segmented. The original segmentations were performed by a radiologist with 5 years of radiology experience who was blinded to post-thrombectomy imaging outcomes. Because the study specifically evaluated the basal ganglia ipsilateral to the occluded vessel, the side of vascular occlusion was disclosed for segmentation purposes only.

### 2.6. Segmentation Reproducibility Analysis

To assess the reproducibility of radiomic features with respect to manual segmentation, 30 cases were randomly selected from the derivation cohort and independently resegmented by a second radiologist with 6 years of radiology experience. The second reader performed manual segmentation on the same 2.5 mm reconstructed axial CT series used for the original analysis and was blinded to the original segmentations, radiomic feature values, and post-thrombectomy imaging outcomes. The side of vascular occlusion was disclosed solely to identify the basal ganglia to be segmented. Radiomic features were subsequently re-extracted from the independently generated VOIs using the same LIFEx preprocessing and feature-extraction settings as in the primary analysis.

Interobserver reproducibility was quantified using a two-way random-effects, absolute-agreement, single-measurement intraclass correlation coefficient [ICC(2,1)]. An ICC threshold of ≥0.80 was used to identify reproducible radiomic features. Only features meeting this criterion were eligible for subsequent feature reduction and model development.

### 2.7. Radiomics Feature Extraction and Preprocessing

Radiomic features were extracted from the segmented VOIs using LIFEx software (version 7.7.0), which implements feature definitions compatible with the Imaging Biomarker Standardization Initiative (IBSI). The same software version and feature-extraction settings were used for the derivation cohort, temporal validation cohort, and interobserver reproducibility analysis. Approximately 182 radiomic outputs were generated, including first-order, shape-based, and texture features.

Radiomic analysis was performed on the 2.5 mm reconstructed axial CT series. For spatial processing in LIFEx, the through-plane voxel spacing was set to 2.5 mm, while the native patient-specific in-plane pixel spacing was retained. When spatial interpolation was required, LIFEx used a fifth-degree Lagrangian polynomial according to the software implementation. No additional spatial filter was applied. Intensity discretization was performed using absolute intensity bounds of −1000 to 3000 HU, with 400 gray levels and a bin size of 10 HU. Texture features were calculated in three dimensions, with a GLCM neighbor distance of 1. The largest connected cluster was retained for texture analysis.

### 2.8. Feature Preprocessing, Selection, and Model Construction

Radiomic outputs that were non-scalar, contained no usable numerical information, or showed no variance within the derivation cohort were excluded before model development. After these exclusions, 141 scalar radiomic features were available for analysis. As an initial reproducibility screen, only features demonstrating adequate interobserver agreement (ICC ≥ 0.80) in the segmentation reproducibility analysis were retained. Of the 141 candidate features, 56 met this reproducibility criterion and were eligible for subsequent model development.

To reduce redundancy and multicollinearity, pairwise Pearson correlation filtering was then applied to the 56 reproducible features within the derivation cohort. For feature pairs with an absolute Pearson correlation coefficient of ≥0.90, redundant features were sequentially removed using a fixed feature-order procedure, leaving 12 non-redundant candidate features. These features were standardized by z-score transformation using means and standard deviations estimated exclusively from the derivation cohort.

Feature selection was subsequently performed using least absolute shrinkage and selection operator (LASSO) logistic regression. The regularization parameter was determined using 10-fold cross-validation and the one-standard-error (1-SE) rule, which selected the most strongly regularized model whose cross-validated loss remained within one standard error of the minimum. This approach was used to favor a parsimonious model and reduce the risk of overfitting in the relatively small derivation cohort. Two radiomic features retained non-zero coefficients at the selected regularization parameter: Gray-Level Run-Length Matrix (GLRLM) Short Runs Emphasis and Gray-Level Size Zone Matrix (GLSZM) Grey Level Variance.

The two selected features were subsequently entered into an unpenalized multivariable logistic regression model fitted in the full derivation cohort. The resulting model constituted the final radiomic model. All feature definitions, preprocessing parameters, standardization parameters, and fitted model coefficients were derived from the derivation cohort and were subsequently fixed for application to the independent temporal validation cohort.

### 2.9. Outcome Definition and Assessment

The primary outcome was post-thrombectomy intracranial hyperdense transformation (HT) identified on follow-up CT. HT was defined as a newly developed hyperdense area within the basal ganglia ipsilateral to the treated arterial territory on the first post-procedural non-contrast CT obtained within 24 h after thrombectomy that was absent on the corresponding preprocedural CT.

Post-thrombectomy CT examinations were evaluated by a single radiologist with 15 years of radiology experience, including 10 years of interventional radiology and 5 years of neurointerventional radiology, without knowledge of the radiomic findings. HT included both contrast extravasation and hemorrhagic transformation. These entities were intentionally analyzed as a unified imaging outcome because the primary objective was to investigate whether preprocedural radiomic characteristics of visually unremarkable basal ganglia were associated with subsequent reperfusion-related hyperdense tissue changes rather than to develop a model specifically for hemorrhagic transformation or symptomatic intracranial hemorrhage.

Although contrast extravasation and hemorrhagic transformation represent distinct radiological and clinical entities, standardized dual-energy CT or systematic 24–48 h follow-up imaging specifically intended to distinguish these entities was not available for all patients. Consequently, reliable retrospective classification of all hyperdense lesions as contrast extravasation versus true hemorrhage was not possible. The combined endpoint should therefore be interpreted as an imaging-based measure of post-reperfusion tissue response rather than as a clinical hemorrhage endpoint.

Additional CT examinations performed during hospitalization because of neurological deterioration or clinical concern were also reviewed. No cases of delayed HT that were absent on the initial follow-up CT were identified.

### 2.10. Model Performance and Temporal Validation

Discriminatory performance of the final radiomic model was assessed using receiver operating characteristic (ROC) analysis and quantified by the area under the ROC curve (AUC). The 95% confidence intervals for AUCs were estimated using stratified nonparametric bootstrap resampling with 10,000 replicates. The classification threshold was determined exclusively in the derivation cohort using the Youden index. Sensitivity, specificity, accuracy, positive predictive value (PPV), and negative predictive value (NPV) were calculated from the corresponding confusion matrix.

Following completion of model development, the model was applied to the independent temporal validation cohort of 31 consecutive eligible patients treated between August and November 2023. The same two radiomic feature definitions, derivation-cohort standardization parameters, fitted logistic regression coefficients, and derivation-derived probability threshold were applied without modification. No feature re-selection, model retraining, recalibration, or threshold optimization was performed in the temporal validation cohort.

### 2.11. Internal Validation and Assessment of Model Optimism

To assess model-development optimism and feature-selection stability, repeated nested cross-validation was performed within the derivation cohort. A stratified five-fold outer cross-validation was repeated 20 times, resulting in 100 outer training-test partitions. Within each outer training set, correlation filtering, feature standardization, LASSO feature selection, and model fitting were performed without access to the corresponding outer test set. LASSO regularization was determined within the outer training data using stratified five-fold inner cross-validation and the 1-SE selection rule described above. Features selected by LASSO within each outer training set were subsequently entered into an unpenalized logistic regression model, which was then applied to the held-out outer test observations.

The ICC-based reproducibility screen was treated as a fixed, outcome-independent prescreen because interobserver segmentations were available for the predefined subset of 30 derivation cases; all subsequent outcome-dependent preprocessing and model-selection steps were repeated exclusively within each outer training set. For each of the 20 repetitions, predictions from the five mutually exclusive outer test folds were combined to obtain out-of-fold predictions for all patients in the derivation cohort, and an AUC was calculated from these predictions. Nested cross-validation performance was summarized as the mean and standard deviation of the 20 repetition-level AUCs.

Feature-selection frequency was additionally recorded across the 100 outer training sets as an indicator of model stability. Nested cross-validation was used solely to characterize internal performance, model optimism, and feature-selection variability and was not used to optimize the model on the basis of temporal-validation performance.

### 2.12. Learning-Curve Analysis

To further examine the relationship between derivation sample size and model performance, a learning-curve analysis was performed for the final two-feature radiomic model. Training and cross-validated AUCs were evaluated at training-set sizes of 20, 30, 40, 50, 60, and 67 patients using repeated stratified five-fold cross-validation with 20 repetitions. Feature standardization was performed within each training fold to avoid information leakage. Mean training and cross-validated AUCs and their standard deviations were calculated at each sample size. This analysis was intended to characterize how training and cross-validated discrimination changed as the number of training observations increased within the available derivation cohort.

### 2.13. Statistical Analysis

Statistical analyses were performed using SPSS software (version 26; IBM Corp., Armonk, NY, USA) and Python (version 3.13.5). Radiomic model development and resampling-based analyses were performed using scikit-learn (version 1.8.0). Normality of continuous variables was assessed using the Shapiro–Wilk test. Normally distributed continuous variables were compared using the independent-samples *t*-test, whereas non-normally distributed variables were compared using the Mann–Whitney U test. Categorical variables were compared using the chi-square test or Fisher’s exact test, as appropriate. Continuous variables are reported as mean ± standard deviation or median (interquartile range), as appropriate, and categorical variables as number and percentage.

For radiomic model development, interobserver reproducibility assessment, correlation filtering, feature standardization, penalized logistic regression, multivariable logistic regression, ROC analysis, repeated nested cross-validation, and learning-curve analysis were performed as described above. The temporal validation cohort was reserved for independent evaluation of the derivation-developed model. All statistical tests were two-sided, and a *p*-value < 0.05 was considered statistically significant for conventional inferential analyses.

## 3. Results

### 3.1. Patient Characteristics

A total of 84 patients who underwent mechanical thrombectomy for acute ischemic stroke were included in the derivation cohort. Of these, 41 patients developed post-procedural intracranial hyperdense transformation (HT), whereas 43 did not.

No statistically significant differences were observed between patients with and without HT with respect to age, ASPECTS, International Normalized Ratio (INR), platelet count, sex distribution, or antithrombotic therapy. Similarly, procedural timing parameters—including symptom-to-groin puncture time, door-to-groin puncture time, and symptom-to-procedure completion time—did not differ significantly between groups (Table 1).

### 3.2. Segmentation Reproducibility

Among the 141 candidate scalar radiomic features, interobserver reproducibility could be evaluated for 108 features. Of these, 56 demonstrated adequate reproducibility (ICC ≥ 0.80) and were retained for subsequent model development, whereas 52 had ICC values < 0.80. ICC could not be meaningfully estimated for the remaining 33 features because of unavailable paired measurements or invariant/non-informative values in the reproducibility subset.

### 3.3. Radiomics Feature Selection and Model Development

After exclusion of non-scalar, non-informative, and invariant radiomic outputs, 141 scalar radiomic features were available for analysis. Following the interobserver reproducibility screen, 56 features with ICC values ≥ 0.80 were retained. Pairwise correlation filtering at an absolute Pearson correlation coefficient threshold of ≥0.90 further reduced the candidate set to 12 non-redundant features.

Following z-score standardization, LASSO logistic regression with 10-fold cross-validation and the 1-SE selection rule retained two features with non-zero coefficients: GLRLM Short Runs Emphasis and GLSZM Grey Level Variance. These two features were subsequently entered into the final unpenalized multivariable logistic regression model (Table 2).

In the final model, higher GLRLM Short Runs Emphasis was associated with increased odds of HT (odds ratio [OR], 2.12; 95% CI, 1.07–4.20; *p* = 0.030). GLSZM Grey Level Variance showed an OR of 1.71 (95% CI, 0.88–3.35; *p* = 0.116).

### 3.4. Model Performance in the Derivation Cohort

**Figure 3:**
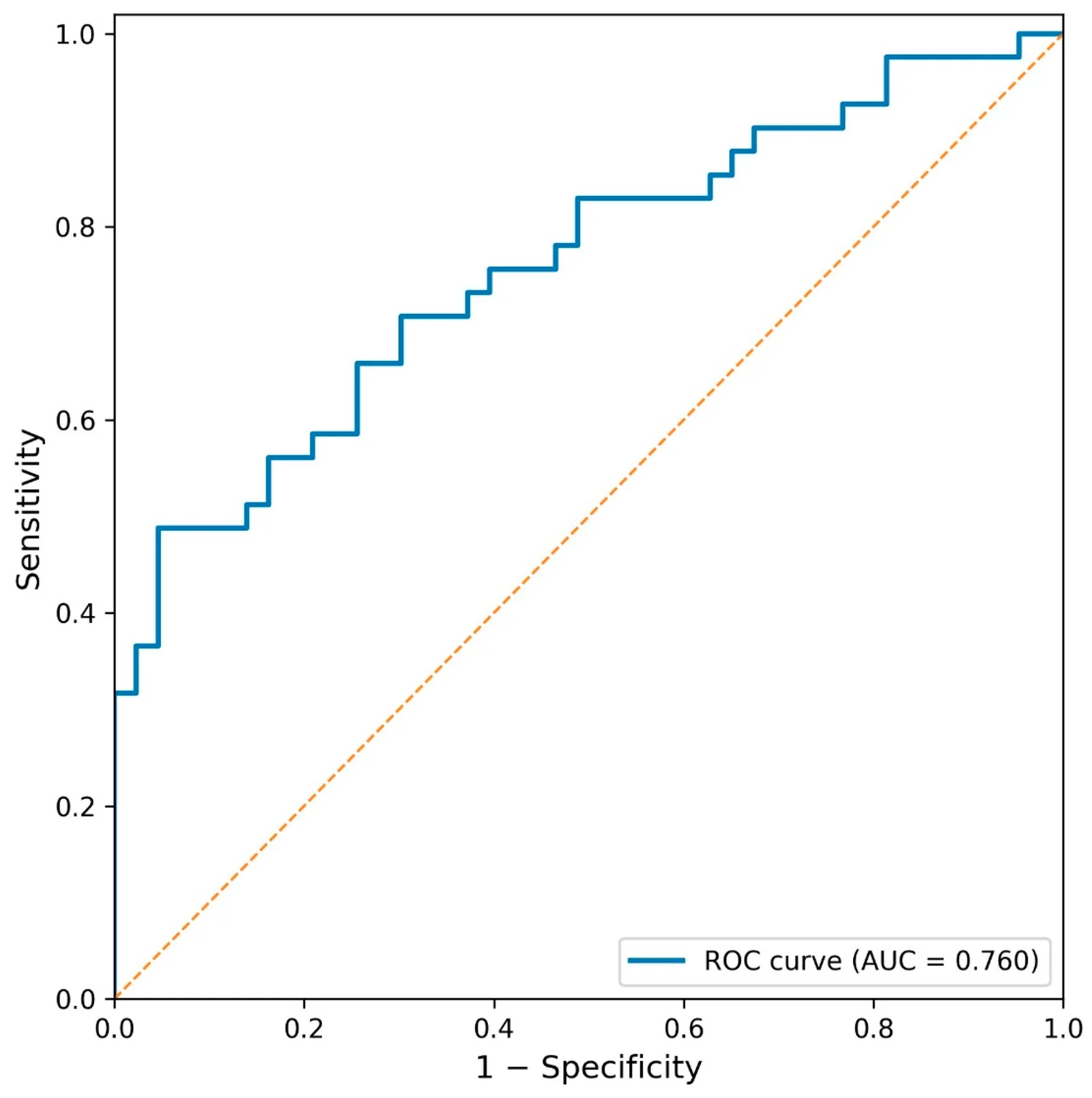
Receiver operating characteristic curve of the final two-feature radiomics model in the derivation cohort. The model achieved an area under the receiver operating characteristic curve (AUC) of 0.760 (95% CI, 0.652–0.855) in the derivation cohort (*n* = 84).

The final two-feature model demonstrated an AUC of 0.760 (95% CI, 0.652–0.855) in the derivation cohort (Figure 3).

The optimal probability threshold determined using the Youden index was 0.672. At this threshold, the confusion matrix consisted of 20 true-positive, 2 false-positive, 41 true-negative, and 21 false-negative classifications. Sensitivity was 48.8%, specificity 95.3%, overall accuracy 72.6%, PPV 90.9%, and NPV 66.1% (Table 3).

### 3.5. Internal Validation and Model Stability

Repeated nested cross-validation yielded a mean AUC of 0.673 ± 0.037 across the 20 repetitions. GLRLM Short Runs Emphasis and GLSZM Grey Level Variance were the most frequently selected features, being retained in 65% and 57%, respectively, of the 100 outer training sets. GLSZM Small Zone Emphasis was selected in 16% of outer models, whereas no other candidate feature was selected in more than 9%.

The median number of features selected per outer model was 2 (IQR, 1–2). In 17 of the 100 outer training sets, the 1-SE rule resulted in no retained radiomic feature, further indicating variability in feature selection across resampled derivation subsets.

### 3.6. Learning-Curve Analysis

**Figure 4:**
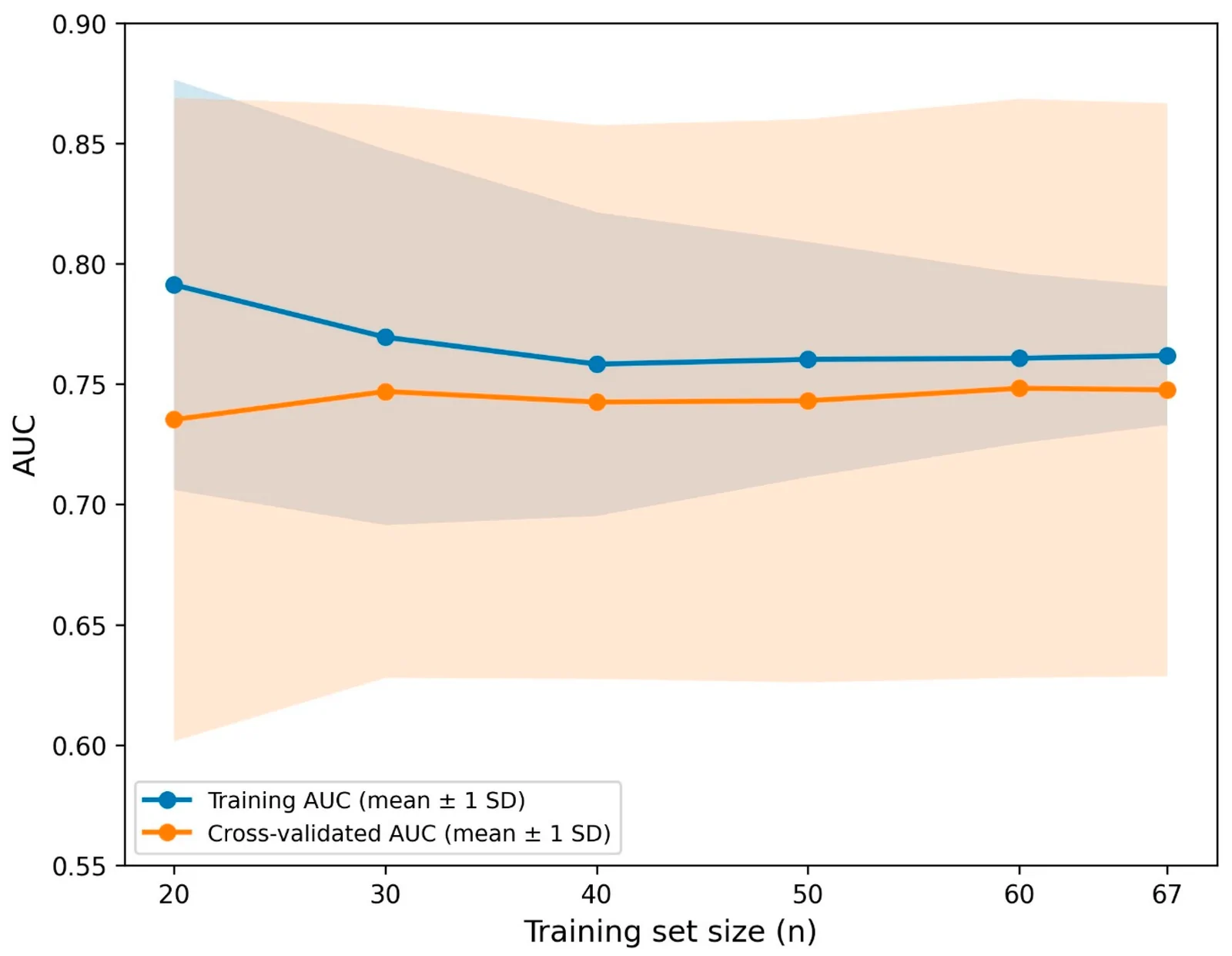
Learning curve of the final two-feature radiomics model. Training and cross-validated AUC values are shown across progressively increasing training-set sizes within the derivation cohort. Performance was evaluated using repeated stratified five-fold cross-validation with 20 repetitions. Solid lines indicate mean AUC values, and shaded areas represent ±1 standard deviation across cross-validation repetitions.

Learning-curve analysis showed relatively limited changes in model performance across increasing training-set sizes. Mean training AUC decreased from 0.791 at a training size of 20 to 0.762 at the largest evaluated training size, whereas mean cross-validated AUC changed only minimally from 0.735 to 0.748. Both curves reached an early plateau, indicating that increasing the training sample within the evaluated range did not materially improve discrimination (Figure 4).

### 3.7. Temporal Validation

The independent temporal validation cohort consisted of 31 eligible patients, including 15 patients with HT and 16 without HT. Baseline demographic and procedural characteristics were descriptively comparable with those of the derivation cohort. Mean age was 64.3 ± 16.4 years in the temporal validation cohort compared with 68.3 ± 14.8 years in the derivation cohort, while median ASPECTS was 10 (IQR, 9–10) in both cohorts. Mean symptom-to-groin puncture times were 222.9 ± 71.9 and 220.6 ± 72.0 min, respectively. Other available baseline and procedural characteristics showed no substantial descriptive differences between cohorts.

**Figure 5:**
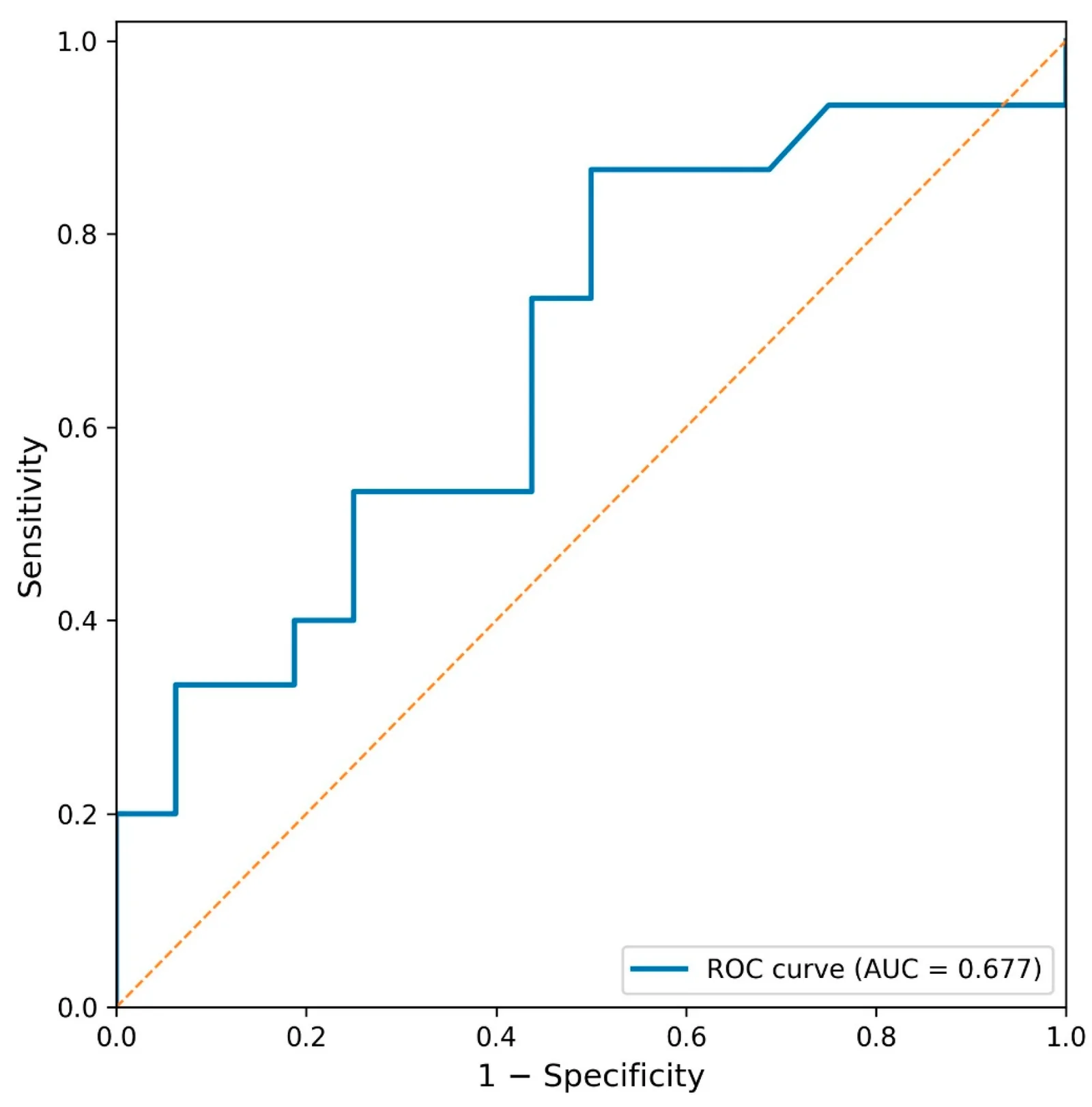
Receiver operating characteristic curve of the locked radiomics model in the independent temporal validation cohort. The model achieved an AUC of 0.677 (95% CI, 0.475–0.858) in the temporal validation cohort (*n* = 31). Model coefficients and preprocessing parameters were derived exclusively from the derivation cohort and applied without model retraining or recalibration.

The derivation-developed two-feature model was applied to the temporal validation cohort without feature re-selection, model retraining, recalibration, or threshold optimization. The model achieved an AUC of 0.677 (95% CI, 0.475–0.858) (Figure 5).

Using the derivation-derived probability threshold of 0.672, the confusion matrix consisted of 5 true-positive, 1 false-positive, 15 true-negative, and 10 false-negative classifications. Sensitivity was 33.3%, specificity 93.8%, overall accuracy 64.5%, PPV 83.3%, and NPV 60.0% (Table 3).

## 4. Discussion

In this study, we investigated whether quantitative radiomic characteristics of visually unremarkable basal ganglia on preprocedural non-contrast CT were associated with subsequent post-thrombectomy hyperdense transformation (HT) in patients with preserved ASPECTS and complete reperfusion. A two-feature radiomic signature comprising GLRLM Short Runs Emphasis and GLSZM Grey Level Variance was identified in the derivation cohort, demonstrating an AUC of 0.760 (95% CI, 0.652–0.855). When applied without modification to the independent temporal validation cohort, the model retained modest discriminatory ability, with an AUC of 0.677 (95% CI, 0.475–0.858). Internal resampling further demonstrated lower and variable discrimination and instability in feature selection, indicating model-development optimism within the relatively small derivation cohort. Accordingly, the present findings should be interpreted as evidence of an exploratory radiomic signal rather than as validation of a clinically established prediction model.

The basal ganglia were selected a priori because of their recognized vulnerability to ischemic and reperfusion-related injury. Deep gray matter structures are supplied predominantly by terminal perforating arteries, have high metabolic requirements, and may be particularly susceptible to microvascular and blood–brain barrier disruption following reperfusion [7,8,41,42,43,44,45,46,47,48,49,50,51]. By restricting analysis to the basal ganglia ipsilateral to the occluded vessel, we sought to evaluate a predefined anatomically and pathophysiologically relevant region rather than performing unrestricted whole-brain feature discovery.

An important aspect of the present study is that all included patients had preserved ASPECTS and no visually appreciable ischemic changes within the segmented basal ganglia on baseline CT. Thus, the radiomic signal was not derived from overt infarction visible on conventional imaging. Instead, the selected features characterized quantitative variations within tissue considered unremarkable by visual assessment. This finding raises the possibility that radiomics may capture subtle quantitative tissue heterogeneity not apparent on conventional visual CT assessment and potentially associated with subsequent tissue response to reperfusion.

The final model retained two texture features: Gray-Level Run Length Matrix (GLRLM) Short Runs Emphasis and Gray-Level Size Zone Matrix (GLSZM) Grey Level Variance. Short Runs Emphasis quantifies the relative prevalence of short runs of similar gray levels and therefore reflects fine-scale textural variation, whereas Grey Level Variance characterizes variability in gray-level intensities across homogeneous zones. Both features had positive coefficients in the final model, indicating that higher feature values were associated with a greater probability of subsequent HT; however, only Short Runs Emphasis reached statistical significance in the final unpenalized logistic regression model. These texture patterns may reflect subtle spatial heterogeneity within visually unremarkable basal ganglia, but radiomic features should not be regarded as direct measures of underlying tissue biology. Their biological substrate cannot be established from the present data, and studies integrating radiomics with perfusion imaging, quantitative blood–brain barrier assessment, or other biological markers are needed to clarify these relationships [20,52,53,54,55,56,57,58,59].

The final model demonstrated an AUC of 0.760 (95% CI, 0.652–0.855) in the derivation cohort and 0.677 (95% CI, 0.475–0.858) in the independent temporal validation cohort. At the probability threshold determined exclusively in the derivation cohort, sensitivity and specificity were 48.8% and 95.3%, respectively, in the derivation cohort and 33.3% and 93.8%, respectively, in temporal validation. Thus, although specificity remained high in both cohorts, sensitivity was limited and decreased further in temporal validation. Importantly, the model coefficients, standardization parameters, and classification threshold were fixed before validation, and no feature re-selection, model retraining, recalibration, or threshold optimization was performed in the validation cohort. The validation AUC suggests that some discriminatory information was retained when the model was transferred unchanged to a temporally separate cohort; however, the reduction in AUC, low sensitivity, and wide confidence interval indicate substantial uncertainty regarding the magnitude and clinical relevance of this signal. These findings therefore do not support use of the current model as a stand-alone clinical prediction tool.

The internal validation findings provide an important counterpoint to the apparent derivation performance. When correlation filtering, standardization, LASSO feature selection, and model fitting were repeated within a nested cross-validation framework, mean discrimination was lower than the apparent derivation performance (AUC, 0.673 ± 0.037). Feature selection also showed instability across resampling iterations: Short Runs Emphasis and Grey Level Variance were selected in 65% and 57% of outer-fold models, respectively, and no feature was selected in 17 of 100 outer-fold models. These findings indicate model-development optimism and limited feature-selection stability, which are important considerations given the high dimensionality of the initial radiomic dataset relative to the derivation sample size.

Learning-curve analysis showed an early plateau, with training AUC decreasing from 0.791 to 0.762 and cross-validated AUC changing only minimally from 0.735 to 0.748 as the training-set size increased. Thus, increasing the number of training observations within the range available in this cohort did not materially improve discrimination, providing little evidence that simply adding similar observations within this sample-size range would substantially improve the performance of the present two-feature model. A plateaued learning curve, however, does not by itself establish whether the observed feature–outcome associations are genuine or spurious; rather, it indicates limited additional performance gain with increasing sample size for the specified model and feature space. Together with the modest nested cross-validated performance and feature-selection instability, this finding further supports a cautious, exploratory interpretation of the model.

The present study was designed to investigate an imaging phenotype rather than clinical outcomes. Accordingly, the current model is not ready for clinical deployment and should not be used to guide patient-level treatment decisions. The reported AUC, sensitivity, and specificity should be interpreted as exploratory performance estimates rather than evidence of established clinical validity. The early plateau of the learning curves, modest nested cross-validated performance, feature-selection instability, and reduced discrimination in temporal validation further emphasize the need for caution. Future studies should evaluate larger multicenter datasets and alternative modeling approaches and should independently assess whether the observed radiomic associations are reproducible before any clinical application is considered.

Contrast extravasation and hemorrhagic transformation were analyzed together as HT in the present study. These entities differ in mechanism, clinical significance, prognosis, and management, and their combination therefore limits interpretation of the endpoint as a hemorrhagic outcome. However, the objective of this study was not specifically to predict symptomatic intracranial hemorrhage or hemorrhagic transformation, but rather to investigate the development of new post-thrombectomy hyperdensity within the basal ganglia as an imaging phenotype of tissue response following reperfusion. Contrast-related and hemorrhagic hyperdensities may both occur in the setting of ischemia-related blood–brain barrier and microvascular injury, although their biological substrates are not identical. Because dual-energy CT was not routinely available and standardized 24–48 h follow-up imaging specifically intended to distinguish contrast staining from hemorrhage was not available for all patients, reliable retrospective classification of all hyperdense lesions into these categories was not possible. Accordingly, the present endpoint should be interpreted as an imaging-based phenotype of post-reperfusion tissue change rather than as a surrogate for clinically significant hemorrhage. Future studies should separately adjudicate contrast staining and hemorrhagic transformation and evaluate established hemorrhage subtypes and symptomatic intracranial hemorrhage as distinct outcomes.

Several previous studies have investigated radiomics for the prediction of hemorrhagic transformation after reperfusion therapies. Many have relied on infarct-core segmentation, perfusion imaging, or multimodal magnetic resonance imaging (MRI) [20,21,24,54,55,56,57]. The present approach differs in focusing specifically on a predefined basal ganglia region that appeared unremarkable on baseline non-contrast CT. This design was intended to investigate whether quantitative information exists within apparently preserved tissue rather than to optimize prediction using all available clinical and imaging variables. Consequently, the present model should not be interpreted as an alternative to established clinical predictors or comprehensive clinical-imaging prediction models. Rather, the findings provide preliminary evidence that region-specific radiomic heterogeneity within visually unremarkable tissue may contain information not captured by conventional visual CT assessment.

The study population was intentionally restricted to patients with ASPECTS ≥8, no visually appreciable basal ganglia abnormality, ICA or M1 occlusion, and complete TICI 3 reperfusion. This selection reduced heterogeneity related to baseline ischemic burden, pre-existing tissue abnormality, vascular territory, and incomplete reperfusion, thereby allowing a more focused evaluation of the relationship between baseline basal ganglia characteristics and subsequent post-reperfusion imaging changes. However, this design necessarily limits generalizability to the broader thrombectomy population. In particular, the present findings cannot be extrapolated to patients with more extensive baseline ischemic changes, M2 or posterior-circulation occlusions, or incomplete reperfusion.

This study has several limitations. First, it was retrospective and conducted at a single center. Although an independent temporal cohort was used for validation, external validation across institutions, scanners, reconstruction algorithms, and treatment protocols was not performed. Second, both the derivation and particularly the validation cohorts were relatively small. The wide confidence interval of the validation AUC, lower performance during nested cross-validation, and variability observed across resampling analyses emphasize the uncertainty surrounding model estimates and the risk of overfitting. Third, feature selection showed limited stability during internal resampling; therefore, the selected two-feature signature should be considered exploratory rather than definitive. Fourth, contrast extravasation and hemorrhagic transformation were analyzed as a combined imaging endpoint because dual-energy CT and standardized 24–48 h follow-up imaging were not available for all patients, precluding reliable retrospective differentiation in every case. Consequently, ECASS hemorrhage subtypes and symptomatic intracranial hemorrhage were not evaluated as separate outcomes. In addition, outcome assessment was performed by a single experienced reader, and interobserver agreement for HT classification was not evaluated. Fifth, the study was designed as an imaging-based analysis rather than a comprehensive clinical prediction study. Variables such as baseline NIHSS, glucose, blood pressure, collateral status, infarct-core volume, number of thrombectomy passes, procedure duration, anesthesia technique, and contrast dose were not incorporated into a comparative clinical model; therefore, the incremental predictive value of radiomics beyond established clinical and procedural factors cannot be determined from the present data. In addition, the present analysis used a LASSO-based feature-selection and logistic-regression framework and did not systematically compare alternative machine-learning classifiers. Given the limited sample size, broader algorithmic comparisons were not pursued because screening multiple classifiers and tuning configurations within the same dataset could further increase model-selection optimism; such comparisons should be evaluated in larger datasets with independent external validation. Sixth, functional outcomes such as modified Rankin Scale scores were not available for analysis, preventing assessment of the relationship between the radiomic signature, HT, and long-term neurological outcome. Seventh, although interobserver reproducibility of radiomic features was formally assessed in a randomly selected subset using independent manual segmentation, manual ROI delineation remains inherently observer-dependent, and a substantial proportion of candidate features did not meet the predefined reproducibility threshold or could not be reliably evaluated. Automated or semi-automated segmentation approaches may improve reproducibility in future studies. Finally, radiomic features are sensitive to image acquisition, reconstruction, and preprocessing strategies. Although acquisition and reconstruction parameters were consistent across the derivation and temporal validation cohorts, multicenter harmonization and independent reproducibility assessment will be required before broader application.

In summary, quantitative radiomic analysis of visually unremarkable basal ganglia on preprocedural non-contrast CT identified an exploratory two-feature imaging signature associated with subsequent post-thrombectomy HT. The model retained modest discriminatory ability when applied without modification to an independent temporal cohort, although performance was lower than in the derivation cohort and internal resampling demonstrated model-development optimism and limited feature-selection stability. These findings suggest that quantitative characteristics of apparently preserved basal ganglia may contain information related to susceptibility to post-reperfusion imaging changes, but the present model should be considered hypothesis-generating. Future studies using larger multicenter datasets, alternative modeling approaches, and independent external validation are needed to determine whether this radiomic signal is reproducible and whether it has potential clinical relevance.

## 5. Conclusions

Preprocedural radiomic analysis of visually unremarkable basal ganglia on non-contrast CT identified an exploratory two-feature imaging signature associated with subsequent post-thrombectomy hyperdense transformation in patients with preserved ASPECTS and complete reperfusion. The model retained modest discriminatory ability when applied without modification to an independent temporal validation cohort, although internal resampling demonstrated model-development optimism and limited feature-selection stability. These findings suggest that quantitative radiomic characteristics of apparently preserved basal ganglia may contain information related to susceptibility to post-reperfusion imaging changes, but the present model should be considered hypothesis-generating. The current model is not ready for clinical deployment and should not be used to guide patient-level treatment decisions. Future studies using larger multicenter datasets, alternative modeling approaches, and independent external validation are required to determine the reproducibility and potential clinical relevance of these findings.

## Figures and Tables

**Table 1:** Comparison of clinical and demographic characteristics of patients based on the presence of intracranial hyperdense transformation after thrombectomy.

Features	No HT (*n* = 43)	HT Present (*n* = 41)	*p*-Value
Age (years)	70.9 ± 14.4 (34–102)	65.6 ± 14.9 (36–87)	0.099
ASPECTS	10 (9–10)	10 (9–10)	0.623 *
INR	1.08 (1.01–1.30)	1.05 (1.01–1.15)	0.363 *
Platelet count (/mm^3^)	234,372 ± 76,756 (61,000–399,000)	232,220 ± 69,120 (137,000–454,000)	0.893
Symptom-to-groin puncture time (min)	225.2 ± 63.7	215.7 ± 80.4	0.551
Door-to-groin puncture time (min)	131.5 ± 53.5	127.9 ± 56.8	0.767
Symptom-to-procedure completion time (min)	256.1 ± 67.6	256.3 ± 81.2	0.987
Male sex, *n* (%)	18 (41.9%)	22 (53.7%)	0.279 **
Antithrombotic therapy, present *n* (%)	25 (58.1%)	15 (36.6%)	0.054 ***

Values are presented as mean ± standard deviation (range), median (interquartile range [IQR]), or number (%), as appropriate. * MannWhitney U test, ** Chi-square test, *** Fisher’s exact test. Other group comparisons were made using independent samples *t*-test. HT: Hyperdense Transformation. ASPECTS: Alberta Stroke Programme Early CT Score. INR: International Normalized Ratio.

**Table 2:** Radiomic features retained in the final multivariable logistic regression model.

Radiomic Feature	Standardized B Coefficient	Odds Ratio (OR) *	95% CI for OR	*p*-Value
GLRLM Short Runs Emphasis	0.753	2.12	1.07–4.20	0.030
GLSZM Grey Level Variance	0.539	1.71	0.88–3.35	0.116

* Odds ratios correspond to a one-standard-deviation increase in the respective radiomic feature because predictors were standardized before model fitting. CI: confidence interval; GLRLM: Gray-Level Run Length Matrix; GLSZM: Gray-Level Size Zone Matrix; OR: odds ratio.

**Table 3:** Performance of the final radiomics model in the derivation and temporal validation cohorts.

Parameter	Derivation Cohort (*n* = 84)	Temporal Validation Cohort (*n* = 31)
AUC (95% CI)	0.760 (0.652–0.855)	0.677 (0.475–0.858)
Probability threshold	0.672	0.672 *
Sensitivity, %	48.8	33.3
Specificity, %	95.3	93.8
Overall accuracy, %	72.6	64.5
PPV, %	90.9	83.3
NPV, %	66.1	60.0
True positive, *n*	20	5
False positive, *n*	2	1
True negative, *n*	41	15
False negative, *n*	21	10

* The probability threshold was determined exclusively in the derivation cohort using the Youden index and applied unchanged to the temporal validation cohort. AUC: area under the receiver operating characteristic curve; CI: confidence interval; PPV: positive predictive value; NPV: negative predictive value.

## Data Availability

The data presented in this study are available on reasonable request from the corresponding author due to patient privacy and ethical restrictions.
